# The Utility of Mesenteric T1 Mapping on MR Enterography in Crohn’s Disease: A Preliminary Study

**DOI:** 10.3390/diagnostics15182293

**Published:** 2025-09-10

**Authors:** Seongkeun Park, Jieun Byun, Youe Ree Kim

**Affiliations:** 1 Machine Intelligence Laboratory, Department of Mechanical Design Engineering, Tech University of Korea, Siheung-si 15073, Republic of Korea; skpark@tukorea.ac.kr; 2 Department of Radiology, College of Medicine, Ewha Womans University, Seoul 07985, Republic of Korea; 3 Department of Radiology, Wonkwang University School of Medicine, Wonkwang University Hospital, Iksan 54538, Republic of Korea; sweetynn@naver.com

**Keywords:** Crohn’s disease, MR enterography, T1 mapping, mesentery

## Abstract

**Background:** Crohn’s disease (CD) is a chronic disorder characterized by transmural bowel wall involvement and mesenteric changes, including inflammation and fibrosis. Although Magnetic Resonance Imaging (MRI)-based scoring systems have been proposed for the quantitative assessment of bowel wall changes in Magnetic Resonance enterography (MRE), there has been limited discussion regarding methods for the quantitative evaluation of mesenteric involvement. T1 mapping is an emerging MRI technique, potentially reflecting inflammation and fibrosis. This study aimed to assess the clinical utility of mesenteric T1 mapping in patients with CD. **Methods:** We retrospectively analyzed 71 adults with CD who underwent MRE from October 2024 to May 2025. Mesenteric native T1, post-contrast T1 relaxation times, their difference (ΔT1), and the extracellular volume (ECV) fraction were measured. Regions of interest were placed in mesenteric tissue adjacent to affected bowel segments, avoiding lymph nodes and artifacts. The Crohn’s Disease Activity Index (CDAI) was used to classify disease activity. Group differences and correlations with CDAI were evaluated. **Results:** Native T1 values were significantly higher in active CD than inactive disease (414.3 ms vs. 355.2 ms, *p* < 0.001). ΔT1 was also elevated in active disease (122.5 ms vs. 55.9 ms, *p* < 0.001), while post-contrast T1 and ECV did not differ significantly. Native T1 and ΔT1 showed significant positive correlations with CDAI (r = 0.53 and r = 0.46, respectively), while ECV had a weaker correlation (r = 0.27, *p* = 0.025). **Conclusions:** Mesenteric T1 mapping shows potential as a non-invasive biomarker of mesenteric involvement in CD. With further validation, mesenteric T1 mapping could enable more comprehensive disease assessment and improve the accuracy of clinical characterization in patients with Crohn’s disease.

## 1. Introduction

Crohn’s disease (CD) is a chronic inflammatory condition that can affect any segment of the gastrointestinal tract [1,2,3]. Inflammation in CD is typically transmural, extending through the full thickness of the bowel wall and often involving the mesentery, leading to complications such as strictures and fistulas [4,5,6].

Magnetic resonance enterography (MRE) is an essential noninvasive tool for assessing CD, offering a detailed evaluation of both bowel wall and extraintestinal features. However, current MRI-based activity indices like the Magnetic Resonance Index of Activity (MaRIA) score focus primarily on mural findings—bowel wall thickness, edema, ulceration, and contrast enhancement—which reflect mucosal inflammation but only indirectly account for mesenteric changes [7,8,9,10]. Extraluminal indicators of active CD, such as mesenteric lymphadenopathy, mesenteric edema, or comb sign, are typically noted qualitatively in radiology reports or included as minor components in certain scoring systems. For example, the MR Enterography Global Score adds modest weighting for the presence of enlarged lymph nodes, fibrofatty proliferation, or a comb sign [7]. Given mounting evidence that mesenteric pathology (creeping, vascular, and lymphatic changes) plays a significant role in Crohn’s disease progression and postoperative recurrence, there is a compelling need for imaging biomarkers that specifically evaluate the mesenteric component of disease activity [6,11,12,13,14,15].

T1 mapping is an emerging MRI technique that enables quantitative, voxel-wise measurement of tissue T1 relaxation times [16,17,18,19]. This technique has been extensively validated in cardiovascular and hepatic imaging and has shown promise for detecting inflammatory and fibrotic changes in soft tissues [20,21,22]. Various techniques exist for measuring T1, including inversion recovery T1 mapping, Look-Locker and modified Look-Locker inversion recovery (MOLLI) sequences, and variable flip angle (VFA) T1 mapping [21,23,24,25]. Among these, the VFA approach, which has been widely used for abdominal T1 mapping, offers a practical balance between accuracy and acquisition time. Beyond cardiovascular and hepatic imaging, T1 mapping has also been applied in other organs. For example, Khorasani et al. demonstrated the utility of the VFA method and reported promising results for T1 mapping in glioma grading [26]. T1 relaxation times are sensitive to alterations in tissue composition, including increased water content, cellular infiltration, and extracellular matrix expansion—all hallmark features of inflamed or fibrotic mesenteric fat. While several studies have evaluated bowel wall T1 mapping in CD, there is limited data regarding its application to mesenteric tissue [27,28]. To our knowledge, the potential utility of T1 mapping in assessing mesenteric inflammation has not previously been investigated.

Therefore, the aim of this study was to assess the clinical utility of mesenteric T1 mapping on MRE in patients with Crohn’s disease.

## 2. Materials and Methods

The retrospective study was approved by the Institutional Review Board of Ewha Woman’s University Medical Center, Seoul Hospital, with the approval number 2025-04-044. Given the retrospective nature of the study, the requirement for informed consent was waived. Between October 2024 and May 2025, 82 consecutive CD patients who underwent MRE with an age of ≥18 years were included. Among them, 11 patients with poor image quality for reliable analysis of mesenteric tissues were excluded from the study population.

### 2.1. MR Imaging Protocol

All MRE examinations were performed using a 3.0T MRI scanner (VIDA, Siemens Healthineers, Erlangen, Germany) with a standardized institutional protocol. Bowel distention was achieved by ingestion of 1000–1500 mL (depending on patient tolerance) of polyethylene glycol solution. The MRI protocol included axial and coronal T2-weighted imaging (HASTE and TrueFISP), diffusion-weighted imaging (DWI), pre- and post-contrast 3D spoiled gradient echo sequences (VIBE), and T1 mapping images. Gadolinium-based contrast (0.1 mmol/kg) was administered intravenously prior to contrast-enhanced sequences. DWI was performed with tridirectional diffusion-sensitizing gradients using two b values (0 and 900 s/mm^2^). The MRI consoles automatically generated ADC maps. Table 1 provides additional acquisition parameters.

T1 mapping was conducted using a 3D VIBE sequence with a dual flip angle (3° and 15°) approach. Images were acquired under breath-hold instructions at two distinct flip angles, enabling calculation of voxel-wise T1 relaxation times based on the variable flip angle (VFA) method. This choice balanced accuracy and clinical feasibility, minimizing scan time and reducing motion-related artifacts, consistent with prior validation studies [29]. T1 maps were reconstructed with vendor-provided software (syngo.Via, MapIt, Siemens Healthineers, Erlangen, Germany). During preprocessing, a retrospective non-rigid motion correction algorithm was applied to reduce misregistration between flip angle acquisitions, and B1 inhomogeneity correction was performed as implemented in the vendor’s reconstruction pipeline

### 2.2. MR Imaging Analysis

MR image analysis was performed by two specialized abdominal radiologists (YRK and JB, each with 15 years of experience interpreting MRE), who were blinded to the clinical data. Regions of interest (ROIs) were manually placed on the adjacent mesenteric tissue of the affected bowel wall on native and post-contrast T1 maps. Post-enhanced T1 maps were obtained from enteric-phase images (~60 s after contrast injection). ROIs were manually drawn in a polygonal shape to include as much of the visible mesentery adjacent to the affected bowel wall as possible on native and post-contrast T1 maps, while carefully excluding visible lymph nodes and artifacts. To standardize the evaluation criteria, both readers initially reviewed the images together and reached a consensus on ROI placement. During this process, all images were visually inspected for artifacts, and datasets with severe misregistration or poor fitting quality were excluded. To minimize recall bias, each reader then performed the analyses independently after a 2-month interval, followed by a repeated evaluation 1 week later. Inter-reader and intra-reader agreements were subsequently assessed based on these independent and repeated measurements.

The extracellular volume (ECV) fraction is a quantitative MRI-derived metric that estimates the proportion of tissue volume occupied by the extracellular space [30,31,32]. It reflects expansion of the extracellular compartment that occurs in response to both acute inflammation and chronic remodeling. We calculated the ECV fraction with the following equation: CV = (1 − hematocrit) × (ΔR1mesentery/Δ R1blood), where R1 = 1/T1 [30].

The difference between the native T1 relaxation estimate and post-enhanced T1 relaxation time was expressed as ΔT1.

The MaRIA score was also calculated for the dominant bowel segment [7]. The MRE variables were obtained, including bowel wall thickness, the presence of ulcers, the presence of mural edema, and enhancement of the bowel wall (relative contrast enhancement, RCE). The calculation formula for RCE was [(SI post-enhance − SI precontrast)/(SI precontrast)] × 100 × (SD noise precontrast/SD noise postenhance). The formula for calculating MaRIA was as follows: MaRIA = 1.5 × wall thickening (mm) + 0.02 × RCE + 5 × edema + 10 × ulcers.

### 2.3. Clinical and Laboratory Data Collection

Patients’ demographics and clinical data were collected from the hospital’s electronic medical record system and included age, sex, and laboratory findings obtained within one week of the MRI examination. Laboratory values included C-reactive protein (CRP) and fecal calprotectin levels. The Crohn’s disease activity index (CDAI) was used to grade disease activity [33]. CDAI < 150 was considered remission, 150–220 was mildly active, 221–450 was moderately active, and ≥450 was severely active.

### 2.4. Statistical Analysis

Continuous variables were reported as means ± standard deviation or median with interquartile range, depending on the distribution. Comparisons between groups were performed using the Kruskal–Wallis test for non-normally distributed variables, followed by Dunn’s post hoc test with Bonferroni correction for multiple comparisons. Correlations between variables were analyzed using Spearman’s rank correlation. Univariable and multivariable linear regressions were also performed with CDAI as the outcome. To avoid perfect multicollinearity (ΔT1 = native_T1 − post-enhanced_T1), two models were fitted: Model 1 included native_T1 and post-enhanced_T1 (excluding ΔT1), and Model 2 included Δ_T1 (excluding native_T1 and post-enhanced_T1). Additionally, to further explore the relative contribution of each variable, we applied machine learning–based models, including random forest, gradient boosting, extra trees, and decision tree methods. For reproducibility analysis, intraclass correlation coefficients (ICCs) were calculated.

All statistical analyses were performed using Python (version 1.5.3), SPSS software version 24 (IBM Corp., Armonk, NY, USA) and MedCalc Statistical Software version 22.009 (MedCalc Software Ltd., Ostend, Belgium). No images or plots were modified by artificial intelligence tools. A two-tailed *p*-value < 0.05 was considered statistically significant.

## 3. Results

A total of 71 patients with Crohn’s disease were included in this study, comprising 53 males (74.6%) and 18 females (25.4%) with a median age of 35 years (IQR 27–44). According to the Montreal classification, most patients were diagnosed between ages 17 and 40 (A2, 77.5%), with 7.0% in A1 (≤16 years) and 15.5% in A3 (>40 years). Disease behavior was heterogeneous, with 38.0% showing B1 (non-stricturing, non-penetrating), 26.8% showing B2 (stricturing), 22.5% showing B3 (penetrating), and 12.7% with perianal disease. The primary disease locations were terminal ileum (L1, 59.2%), colon (L2, 4.2%), and ileocolic involvement (L3, 36.6%). The median CDAI ranged broadly (15–184), with 62.0% categorized as inactive and 38.0% as active, including mildly active (19.7%) and moderately active (18.3%) subgroups (Table 2).

### 3.1. MR Parameters of Bowel Wall and Mesentery

**Figure 1:**
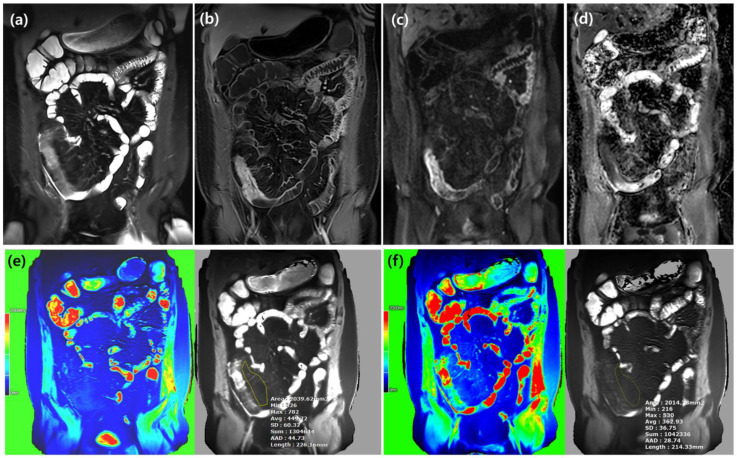
Images from a patient with moderately active inflammatory Crohn’s disease (CDAI = 385.1). The thickened terminal ileum exhibits mural and perienteric edema (**a**), mural enhancement, and prominent vasa recta (**b**). Restricted diffusion is seen on DWI b-900 (**c**) and ADC map (**d**). The coronal native T1 map (**e**) demonstrates a T1 relaxation time of 449.7 ms, exceeding the median native T1 value reported for active disease patients. Post-enhanced T1 map (**f**) demonstrates a T1 relaxation time of 362.9ms. ΔT1 was 86.8 ms.

**Figure 2:**
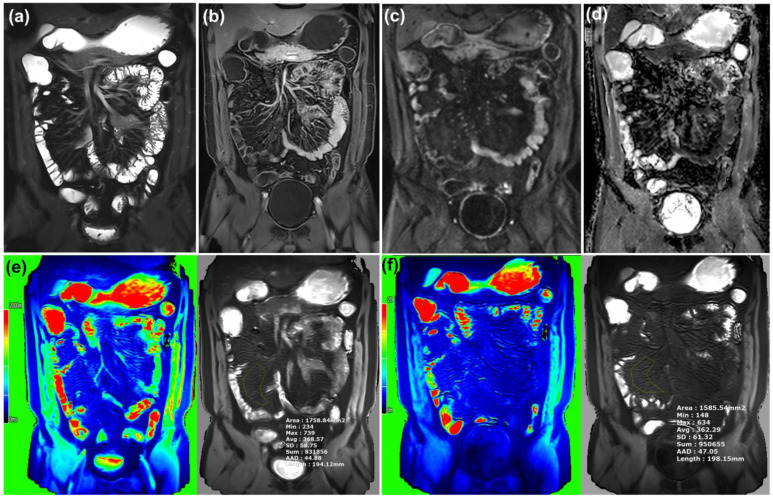
Images from a patient with inactive Crohn’s disease (CDAI = 31.1). No significant imaging sign of active bowel inflammation on T2WI (**a**), postcontrast T1WI (**b**), DWI b-900 (**c**), and ADC (**d**). The coronal native T1 map (**e**) demonstrates a T1 relaxation time of 368.6 ms. Post-enhanced T1 map (**f**) demonstrates a T1 relaxation time of 362.3 ms. ΔT1 was 6.3 ms.

Table 3 shows the comparison of quantitative MRI analysis between inactive and active Crohn’s disease. Native T1 relaxation estimates were significantly higher in active disease (median = 414.3 ms, IQR = 363.4–467.9, Figure 1) than in inactive patients (355.2 ms, 314.9–394.9; *p* < 0.001, Figure 2). Post-enhanced T1 relaxation estimates did not significantly differ between inactive (294.5 ms, 231.2–344.3) and active groups (298.7 ms, 232.3–363.0; *p* = 0.5). However, the ΔT1 was significantly higher in active disease (122.5 ms, 59.9–174.9) than in inactive patients (55.9 ms, 26.2–104.1; *p* < 0.001). The ECV fraction trended higher in active disease (15.7%, 7.3–31.0) than in inactive (9.7%, 3.8–19.4; *p* = 0.1), though this did not reach statistical significance. As a bowel wall characteristic, the MaRIA score was markedly elevated in active disease (26.9, 19.7–34.1) compared to inactive patients (6.1, 4.5–17.1; *p* < 0.001).

### 3.2. Subgroup Analysis of MRI Parameter of Bowel Wall and Mesentery

**Figure 3:**
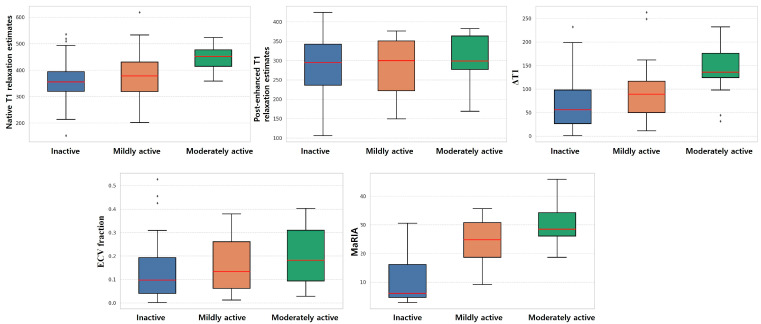
Boxplot of the T1 relaxation estimates related parameters by activity.

In subgroup analysis (Table 4), native T1 relaxation estimates increased progressively from inactive (median = 374.2 ms, IQR = 332.9–434.6) to mildly active (377.8 ms, 295.8–441.4) and moderately active groups (451.9 ms, 407.6–486.1). Significant differences were observed between inactive and moderately active groups (*p* < 0.001), and between mild and moderate activity (*p* = 0.038). The difference between native and post-enhanced T1 relaxation times was also greater in more active disease states. Inactive patients showed a median difference of 73.5 ms (39.4–138.2), compared to mildly active patients (89.3 ms, 39.7–132.3; *p* = 0.245) and moderately active patients (135.9 ms, 111.1–194.1; *p* = 0.002). ECV fraction values showed a similar increasing trend: from 11.4% in inactive, to 13.4% in mildly active, and 18.2% in moderately active groups. While pairwise differences did not all reach statistical significance (*p* > 0.07, all). Post-enhanced T1 values, in contrast, did not show consistent or significant differences between groups (overall *p* = 0.677). MaRIA scores mirrored T1-based measurements in mesentery, increasing from a median of 16.4% in inactive disease to 24.7% in mildly active and 28.5% in moderately active groups, albeit no significant difference between mildly and moderately active disease (inactive vs mildly active and mildly vs. moderately, *p* > 0.001; mildly vs moderately, *p* = 0.105). These findings are illustrated in Figure 3, where native T1 relaxation estimate, ΔT1, and MaRIA displayed clear increasing trends across activity groups. In contrast, the post-enhanced T1 relaxation estimate and the ECV fraction showed overlapping distributions with no discernible separation between groups.

### 3.3. Correlation Analysis Between Parameters

**Figure 4:**
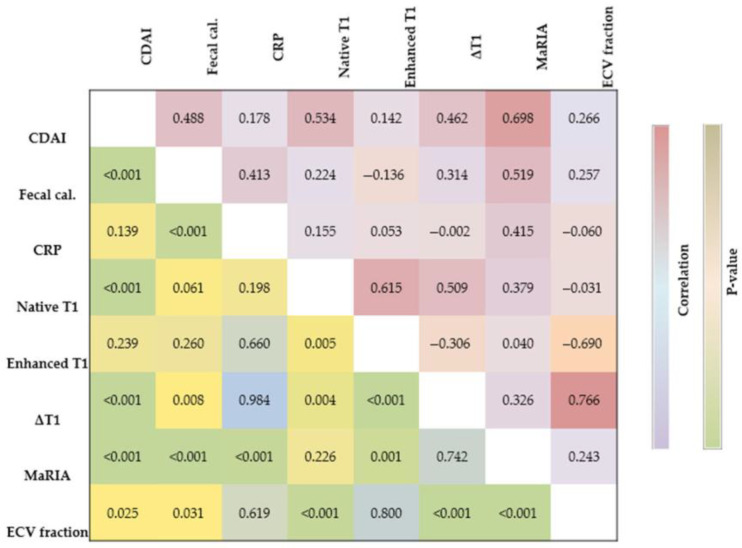
Correlation matrix of the study parameters. Spearman correlation coefficients were used, with the upper triangle displaying correlation coefficients and the lower triangle showing corresponding *p*-values. Fecal cal., fecal calprotectin; Enhanced T1, post-enhanced T1; MaRIA, Magnetic Resonance Index of Activity.

**Figure 5:**
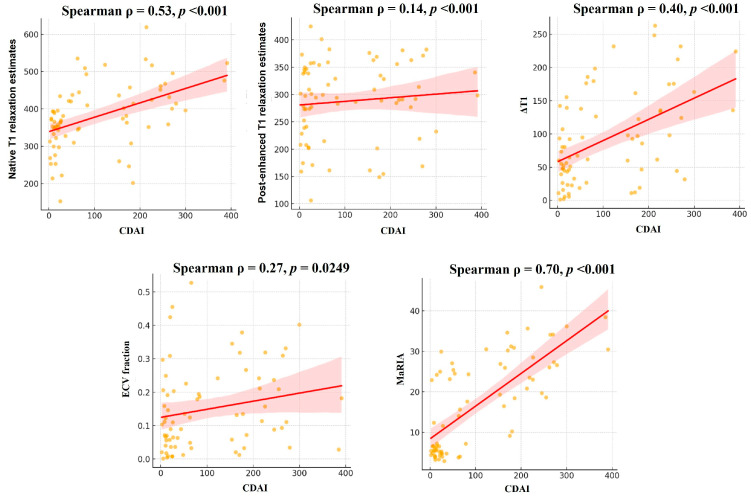
Scatter plot with regression line showing the relationship between CDAI and the MR parameter.

Figure 4 and Figure 5 represent the association between disease activity and MRI parameters. The native T1 relaxation estimates had significant positive correlations with CDAI scores (r = 0.53, *p* < 0.001). The ΔT1 similarly correlated with CDAI (r = 0.46, *p* < 0.001). ECV fraction demonstrated moderate correlations with CDAI (r = 0.27, *p* = 0.025). Among MRI parameters, MaRIA showed a strong positive correlation with CDAI (r = 0.70, *p* < 0.001).

In regression analyses, including CRP, fecal calprotectin, and the MaRIA score together with T1 parameters (i.e., Native T1 relaxation time, ΔT1, and ECV fraction), the MaRIA score emerged as the strongest independent predictor of CDAI. T1 parameters showed modest correlations in univariable analyses but, except for ΔT1 in model 2, were not independently significant after adjustment (Table 5). In an exploratory analysis using machine learning–based models, similar results were obtained. Detailed results of these models are provided in the Supplementary Material (Supplementary Result and Figure S1).

### 3.4. Reproducibility of T1 Mapping Measurement

Intra- and interobserver reproducibility for mesenteric MR parameters was good to excellent (Table 6). For native T1 relaxation estimates, intraobserver ICCs were 0.92 (95% CI: 0.86–0.95) and 0.85 (95% CI: 0.70–0.93), with an interobserver ICC of 0.81 (95% CI: 0.69–0.88). For post-enhanced T1 relaxation estimates, intraobserver ICCs were 0.73 (95% CI: 0.58–0.83) and 0.84 (95% CI: 0.69–0.92), with an interobserver ICC of 0.74 (95% CI: 0.59–0.84). For ECV fraction, intraobserver ICCs were 0.85 (95% CI: 0.75–0.91) and 0.81 (95% CI: 0.63–0.91), with an interobserver ICC of 0.76 (95% CI: 0.62–0.85).

## 4. Discussion

In this preliminary study, we assessed the mesenteric T1 mapping parameters and investigated the association between those parameters and disease activity in patients with CD. To our knowledge, this is the first study to apply T1 mapping specifically to the mesentery in CD, thereby providing novel insights into the potential utility of T1 mapping for understanding mesenteric changes associated with CD. Our results demonstrate that native T1 and ΔT1 were elevated in active disease, with consistent correlations with clinical indices such as CDAI.

T1 mapping is a novel quantitative MR technique measuring the intrinsic tissue T1 relaxation estimates, providing more objective characterization of tissue properties [19,34]. T1 mapping has been widely investigated in cardiac and hepatic MRI, with growing interest in its application to characterize other organs, such as the bowel wall and kidneys [28,35,36,37]. While ECV, a combined parameter of native and contrast-enhanced T1 mapping, as well as post-contrast T1 values, primarily reflect changes in the extracellular space, native T1 relaxation measurement has been shown to reflect both intracellular and extracellular/interstitial factors. This characteristic also represents an advantage of T1 mapping in tissue pathology assessment [19]. There is considerable evidence that T1 mapping indices are affected by myocardial edema in settings such as acute myocardial infarction, acute or chronic myocarditis, and Takotsubo cardiomyopathy, as demonstrated by cardiac MRI [32,38,39,40]. T1 mapping has demonstrated superior performance compared with T2-weighted imaging for detecting acute inflammation [32,40,41]. Similar to the results of these studies, our findings highlight the potential of T1 mapping for detailed characterization of mesentery in CD patients, offering a more comprehensive evaluation of disease activity. Elevated native T1 relaxation estimates in active disease likely reflect increased mesenteric edema and water content due to inflammatory infiltration. The ΔT1 was also significantly greater in active disease, indicating more prominent contrast agent uptake within the extracellular space, a hallmark of increased vascular permeability and inflammation.

While the ΔT1 provided clear separation between activity grades, post-enhanced T1 values alone did not significantly differentiate active from inactive disease. This suggests that dynamic changes in T1 following contrast administration better capture mesenteric permeability and extracellular expansion [42,43]. A previous study on cardiac MRI using T1 mapping showed that the post-enhanced T1 relaxation estimate is influenced by several confounding factors such as contrast dose, clearance rate, scan timing, body composition, and hematocrit, which leads to significant variations in the post-contrast T1 maps [42,43,44]. To minimize such influence, post-contrast T1 maps in our study were consistently acquired during the enteric phase according to standardized MRE protocols. This approach was intended to reduce, but may not completely eliminate, inter-patient differences related to contrast kinetics and hemodynamics. Moreover, post-contrast tissue T1 is also affected by the baseline native T1 properties of the tissue; for instance, preexisting tissue characteristics like fibrosis or fat content can alternative T1 values and potentially mask or blunt the specific T1 shortening due to acute inflammatory enhancement [44]. Considering that even patients with inactive Crohn’s disease may have ongoing mesenteric fibrosis, we believe that this factor may also have contributed to our results.

Although the ECV fraction correlated with CDAI, it did not significantly differentiate active from inactive disease. This discrepancy may be due to the considerable overlap in ECV values between groups, the heterogeneity of the active group, and the fact that ECV reflects not only acute inflammatory changes but also chronic fibrotic remodeling. As a result, some inactive patients may retain elevated ECV values despite clinical remission, reducing the discriminatory power of group-based comparisons [45,46]. The ECV fraction is known to reflect interstitial volume expansion, which encompasses both acute inflammatory edema and chronic fibrotic remodeling [46]. In Crohn’s disease, it is well recognized that mesenteric abnormalities can persist even during remission; for example, “creeping fat” (the expansion of mesenteric adipose tissue encasing the bowel) and mesenteric fibrotic thickening often remain in patients with longstanding disease [3,11,47,48]. Such chronic changes could keep the mesenteric ECV fraction high in clinically inactive patients, thereby masking the differences that would be expected from inflammation alone.

Importantly, T1 mapping and the ECV fraction have been well established in cardiac MR imaging, demonstrating strong correlations with histopathologic findings across a variety of diseases [49,50]. This supports the notion that T1 values and ECV fraction can sensitively reflect tissue-level cellular changes. Such sensitivity suggests their potential utility in capturing the diverse pathophysiological alterations of the mesentery in heterogeneous diseases like Crohn’s disease. However, this study was a preliminary investigation focused solely on assessing MR parameter changes in the mesentery according to clinical disease activity. As such, the absence of a statistically significant difference in the ECV fraction observed across activity levels may reflect this limitation. Future studies incorporating mesenteric pathology specimens could enable stratified analyses of MR parameters, distinguishing inflammatory from fibrotic components, and ultimately facilitate the development of MR biomarkers capable of capturing heterogeneity in Crohn’s disease.

In our regression analysis, the MaRIA score demonstrated the strongest association with CDAI. This finding is not surprising, given that MaRIA was originally developed and has been extensively validated as an imaging-based index specifically designed to assess Crohn’s disease activity [7,8,9]. In contrast, T1 parameters showed relatively weaker associations with CDAI. This result may be explained by the fact that T1 relaxation estimates reflect not only inflammatory changes but also fibrotic alterations within the tissue [38]. Therefore, while MaRIA closely parallels clinical disease activity by design, T1 mapping may provide complementary information by capturing both acute inflammation and chronic structural changes.

Reproducibility analysis demonstrated good to excellent intra- and interobserver agreement for mesenteric T1 mapping and ECV fraction, supporting the reliability of these quantitative measurements.

Beyond T1 mapping, other MR techniques may also provide complementary information for mesenteric evaluation. Traditionally, diffusion-weighted imaging (DWI) and apparent diffusion coefficient (ADC) mapping have been recognized as valuable tools for detecting active inflammation in Crohn’s disease [51]. While the importance of DWI in bowel wall assessment is well established, its role in evaluating mesenteric involvement has not been sufficiently investigated. Given that DWI reflects water content and tissue edema [52], it has the potential to enhance diagnostic assessment of mesenteric changes. Future studies incorporating DWI for mesenteric evaluation may therefore provide complementary insights into disease activity and broaden the scope of imaging biomarkers in Crohn’s disease.

Despite these promising findings, our study has limitations. This study was conducted in a single center with a relatively small sample size. In particular, no severely active disease patients were included in the subgroup analysis. This may have limited the power to detect significant differences for parameters. Future studies should include larger and multicenter cohorts. We also lacked histological correlation of mesenteric tissue, preventing direct validation of imaging findings against tissue-level inflammation or fibrosis. Incorporating pathologic results, combining T1 mapping with other imaging modalities, such as diffusion-weighted imaging, elastography, or dynamic contrast-enhanced MRI, could offer a more comprehensive characterization of mesenteric inflammation and fibrosis. Medication use, including corticosteroids, immunomodulators, and biologics, may also have influenced both disease activity and T1 values by reducing inflammation or altering tissue properties. Although we provided an overview of therapies at the time of imaging, residual confounding by treatment cannot be excluded. Lastly, while we attempted to standardize ROI placement and avoid vascular or lymphatic structures, the inherent heterogeneity of mesenteric tissue and motion artifacts in the abdomen may have introduced variability.

Despite these limitations, our findings suggest possible clinical implications that warrant further investigation. This study was conceived with a particular interest in the mesenteric environment, which is increasingly recognized as playing a role in Crohn’s disease progression and complications. Our study was not intended to position mesenteric T1 mapping as a direct replacement for established indices such as MaRIA, which consistently emerged as the strongest predictor of disease activity. However, T1 mapping captured distinct tissue-level information by reflecting not only active inflammation but also fibrotic remodeling within the mesentery, thereby offering complementary insights not fully represented by inflammation-focused indices. Quantitative T1 parameters could potentially assist in monitoring treatment response, guiding therapeutic decisions, and predicting long-term outcomes. From a practical standpoint, T1 mapping requires only a short additional acquisition and simple post-processing, suggesting that its integration into routine protocols may be feasible. Nevertheless, these considerations remain preliminary, and validation in larger multicenter cohorts with standardized protocols will be essential before clinical application can be realized.

## 5. Conclusions

In conclusion, our preliminary findings suggest that T1 mapping holds potential as a non-invasive marker for assessing mesenteric involvement in patients with Crohn’s disease. Native T1 relaxation estimates, the difference between native and post-enhanced T1, and the ECV fraction reflect mesenteric tissue characteristics that are associated with inflammatory burden. With further validation, mesenteric T1 mapping could enable more comprehensive disease assessment and improve the accuracy of clinical characterization in patients with Crohn’s disease.

## Figures and Tables

**Table 1:** MRE parameters.

	T2 HASTE Coronal	T2 HAST Coronal (Fat Suppression)	T2 Truefisp Coronal (Fat Suppression)	T2 Truefisp Axial (Fat Suppression)	DWI	Coronal Gb-Enhanced 3D T1 Weighted VIBE
Repetition time (ms)	1000	1000	520.85	300.8	2000	3.95
Echo time (ms)	81	79	2	2	49	1
Image matrix	384 × 307	320 × 320	256 × 154	256 × 156	100 × 130	320 × 260
Flip angle	137	170	44	51	90	9
Section thickness (mm)	5.0	5.0	5.0	5.0	5.0	2.5
Section gap (mm)	0.5	0.5	0.0	0.0	0.0	0.0

HASTE = half-Fourier acquisition single-shot turbo spin-echo. The HASTE sequences were performed with and without fat suppression (FS). bTrue FISP = true fast imaging with steady-state precession. VIBE = volume interpolated breath-hold.

**Table 2:** The demographic and clinical characteristics of patients with Crohn’s disease.

Variables		Total (*n* = 71)
Age (y), mean ± SD)		37.1 ± 12.5
sex *n* (%)	male	53 (74.6)
	female	18 (25.4)
Montreal classification		
Age at diagnosis *n* (%)	A1 (≤16)	5 (7.0)
	A2 (17–40)	55 (77.5)
	A3 (>40)	11 (15.5)
Behavior, *n* (%)	B1-non-strictureingm non-penetrating	27 (38.0)
	B2-stricturing	19 (26.8)
	B3-penetrating	16 (22.5)
	p	9 (12.7)
Location, *n* (%)	L1-terminal ileum	42 (59.2)
	L2-colon	3 (4.2)
	L3-ileocolic	26 (36.6)
Current medication, *n* (%)	Anti-inflammatory drug	8 (11.3) *
	Immunomodulator	21 (29.6) *
	Biologics	42 (59.2) *
CDAI, median (IQR)		54 (15, 184)
Fecal calprotectin, median (IQR)		110.0 (19.5, 651.0)
CRP, median (IQR)		0.10 (0.03, 0.33)

* Percentages may not total 100% due to rounding. CDAI, Crohn’s disease activity index; CRP, C-reactive protein.

**Table 3:** MRI parameters of the bowel wall and mesentery in patients with inactive and active Crohn’s Disease.

	All	Inactive (*n* = 44)	Active (*n* = 27)	*p*-Value
Native T1 relaxation estimates (ms)	374.2 (332.9, 434.6)	355.2 (314.9, 394.9)	414.3 (363.4, 467.9)	<0.001
Post-enhanced T1 relaxation estimates (ms)	294.6 (232.3, 347.1)	294.5 (231.2, 344.3)	298.7 (232.3, 363.0)	0.546
ΔT1 (ms)	73.5 (39.4, 138.2)	55.9 (26.2, 104.1)	122.5 (59.9, 174.9)	0.008
ECV fraction (%)	11.4 (4.9, 22.6)	9.7 (3.8, 19.4)	15.7 (7.3, 31.0)	0.066
MaRIA	16.4 (5.4, 26.6)	6.1 (4.5, 17.1)	26.9 (19.7, 34.1)	<0.001

Data are expressed as median with interquartile range. ΔT1, difference between native and post-enhanced T1 relaxation estimates; ECV, extracellular volume; MaRIA, Magnetic Resonance Index of Activity.

**Table 4:** Subgroup analysis of MRI parameters of the bowel wall and mesentery by activity grade.

	Inactive (*n* = 43)	Mildly Active (*n* = 14)	Moderately Active (*n* = 13)	*p*-Value	Inactive vs. Mildly	Inactive vs. Moderate	Mildly vs. Moderate
native T1 relaxation estimates (ms)	374.2 (332.9, 434.6)	377.8 (295.8, 441.4)	451.9 (407.6, 486.1)	0.001	0.414	<0.001	0.038
Post-enhanced T1 relaxation estimates (ms)	294.6 (232.3, 347.1)	299.7 (191.6, 357.6)	298.7 (254.7, 367.4)	0.677	0.877	0.413	0.458
ΔT1 (ms)	73.5 (39.4, 138.2)	89.3 (39.7, 132.3)	135.9 (111.1, 194.1)	0.007	0.245	0.002	0.048
ECV fraction (%)	11.4 (4.9, 22.6)	13.4 (5.2, 28.0)	18.2 (9.1, 31.4)	0.155	0.261	0.074	0.616
MaRIA	16.4 (5.4, 24.7)	24.7 (17.9, 31.0)	28.5 (24.6, 35.2)	<0.001	<0.001	<0.001	0.105

Data are expressed as median with interquartile range. Group differences were tested using the Kruskal–Wallis test, followed by Dunn’s post hoc test with Bonferroni correction. ECV, extracellular volume; MaRIA, Magnetic Resonance Index of Activity.

**Table 5:** Univariable and multivariable linear regression analyses with CDAI as the dependent variable.

	Univariable					
Variable	B (SE)	*p*-value	95% CI			
MaRIA	6.68 (0.71)	<0.001	5.28–8.07			
native T1 relaxation estimates	0.56 (0.13)	<0.001	0.30–0.83			
ΔT1	0.76 (0.16)	<0.001	0.45–1.07			
post-enhanced T1 relaxation estimates	0.14 (0.17)	0.39	−0.18–0.46			
ECV fraction	174.76 (109.78)	0.11	−40.42–389.93			
Fecal calprotectin	0.04 (0.02)	0.02	0.01–0.08			
CRP	22.38 (27.08)	0.41	−30.69–75.44			
	**Multivariable**					
	**Model 1 (R^2^ = 0.61)**			**Model 2 (R^2^ = 0.60)**		
Variable	B (SE)	*p*-value	95% CI	B (SE)	*p*-value	95% CI
MaRIA	5.29 (1.02)	<0.001	3.29–7.29	5.55 (1.04)	<0.001	3.53–7.58
native T1 relaxation estimates	0.36 (0.22)	0.11	−0.08–0.79			
ΔT1				0.45 (0.20)	0.02	0.06–0.83
post-enhanced T1 relaxation estimates	−0.09 (0.34)	0.79	−0.77–0.58			
ECV fraction	76.11 (136.42)	0.58	343.48–0.09	−75.40 (99.13)	0.45	−269.69–118.88
Fecal calprotectin	0.00 (0.02)	0.98	−0.03–0.03	0.00 (0.02)	0.97	−0.03–0.03
CRP	10.23 (11.70)	0.38	−12.71–33.17	8.08 (12.33)	0.51	−16.08–32.24

Note. Model 1, ΔT1 was excluded due to multicollinearity; Model 2, native T1 relaxation estimates and post-enhanced T1 relaxation estimates were excluded due to multicollinearity.

**Table 6:** Intraclass correlation coefficient (ICC) scores for intra- and inter-observer agreement for measuring T1 parameters.

	Intraobserver (95%CI)		Interobserver (95%CI)
	**Observer 1**	**Observer 2**	
native T1 relaxation estimates	0.92 (0.86–0.95)	0.85 (0.70–0.93)	0.81 (0.69–0.88)
post-enhanced T1 relaxation estimates	0.73 (0.58–0.83)	0.84 (0.69–0.92)	0.74 (0.59–0.84)
ECV fraction	0.85 (0.75–0.91)	0.81 (0.63–0.91)	0.76 (0.62–0.85)

## Data Availability

The original contributions presented in this study are included in the article. Further inquiries can be directed to the corresponding author.

## Supplementary Materials

The following supporting information can be downloaded at: https://www.mdpi.com/article/10.3390/diagnostics15182293/s1, Figure S1: Relative feature importance derived from various machine learning–based regression models.

## Abbreviations

The following abbreviations are used in this manuscript:

CD	Crohn’s disease
MRE	Magnetic resonance enterography
ECV	extracellular volume
CDAI	Crohn’s Disease Activity Index
