# Supplementary material for: The Utility of Mesenteric T1 Mapping on MR Enterography in Crohn’s Disease: A Preliminary Study
Source: Diagnostics (Basel). 2025 Sep 10;15(18):2293. doi: 10.3390/diagnostics15182293 (PMC12468882; doi:10.3390/diagnostics15182293)
Supplement: Supplementary file 1 [file diagnostics-15-02293-s001.zip › diagnostics-3803662-supplementary.pdf]

diagnostics

MDPI

Supplementary Materials

Supplementary result

To further explore variable contributions, we also applied machine learning–based models (ie. random forest, gradient boosting, extra trees, and decision tree). The feature importance profiles from these models were consistent with the regression findings, highlighting the predominant role of MaRIA and indicating complementary, albeit weaker, contributions of T1 parameters (Supplementary Figure S1).

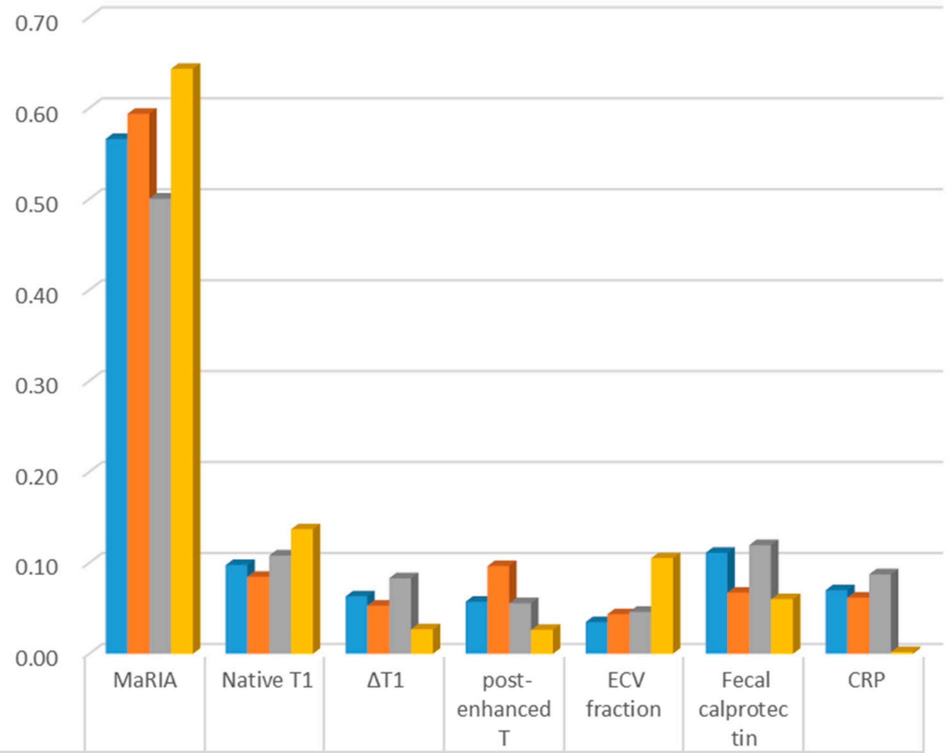

	MaRIA	Native T1	ΔT1	post-enhanced T	ECV fraction	Fecal calprotectin	CRP
Random Forest Regression((R^2=0.928)	0.57	0.10	0.06	0.06	0.03	0.11	0.07
Gradient Boosting Regression (R^2=0.996)	0.59	0.08	0.05	0.10	0.04	0.07	0.06
Extra Trees Regression (R^2=1)	0.50	0.11	0.08	0.06	0.05	0.12	0.09
Decision Tree Regression (R^2=1)	0.64	0.14	0.03	0.03	0.11	0.06	0.00

Figure S1. Relative feature importance derived from various machine learning–based regression models.

 https://doi.org/10.3390/diagnostics15182293
